# Characterizing dissimilarity of weighted networks

**DOI:** 10.1038/s41598-021-85175-9

**Published:** 2021-03-11

**Authors:** Yuanxiang Jiang, Meng Li, Ying Fan, Zengru Di

**Affiliations:** School of Systems Science, Beijing Normal University, Beijing, 100875 China

**Keywords:** Statistical physics, thermodynamics and nonlinear dynamics, Complex networks

## Abstract

Measuring the dissimilarities between networks is a basic problem and wildly used in many fields. Based on method of the D-measure which is suggested for unweighted networks, we propose a quantitative dissimilarity metric of weighted network (WD-metric). Crucially, we construct a distance probability matrix of weighted network, which can capture the comprehensive information of weighted network. Moreover, we define the complementary graph and alpha centrality of weighted network. Correspondingly, several synthetic and real-world networks are used to verify the effectiveness of the WD-metric. Experimental results show that WD-metric can effectively capture the influence of weight on the network structure and quantitatively measure the dissimilarity of weighted networks. It can also be used as a criterion for backbone extraction algorithms of complex network.

## Introduction

Since various systems with complex interactions can be abstractly represented as networks, network science has developed rapidly and widely used in various fields such as biology^1–3^, economics^4,5^ and social science^6–8^. One of the most important features of network science is that it can extract the common characteristics of different systems under the network representation. The most representative is the study about the nontrivial topological properties such as community structure and long-tail degree distribution. Therefore, how to accurately extract network topological characteristics and find out the general rules of different systems is the focus and difficulty of network science^9–11^.

About network topologies, many scholars have shown great interest in comparison of complex networks^1,2,12,13^, which is mainly to measure the differences between two networks by comparing their topological properties. Network comparison is the basic of many network analysis applications such as model selection^14^, network classification and clustering^15^, anomaly and discontinuity detection^16^, and evaluation of sampling algorithms^17^. The core of network comparison is to define an effective dissimilarity metric^18–20^, which can capture and adequately quantify topological differences between networks even when they have different sizes. Moreover, a good dissimilarity metric should have the ability to recognize the different roles of links and nodes, considering overall structural properties.

The network comparison comes from the graph comparison in graph theory. Early graph comparison methods, such as graph isomorphism^21,22^ and edit distance^23–25^, are mainly based on graph matching^26^ technology to decide whether two graphs are identical. Generally, the algorithms have the time complexity of NP-Complete so that they are time-consuming for large networks and could only work on graphs with relatively few nodes. Vishwanathan and Kondor et al. put forward the Graph Kernels, which decomposes the graph into multiple substructures and then determines whether substructures are isomorphic^27^. This method has the obvious advantage of reducing the time complexity from NP-Complete to polynomial level, but the kernel function is difficult to construct. Mieghem et al. used the eigenvalue vector of the graph’s adjacency matrix or Laplacian matrix to represent the network structure and characterized the graph’s distance by comparing the differences between the two vectors^28^. This method is relatively simple to understand and operate, but it is only applicable to the comparison of two graphs with the same number of nodes, and it cannot accurately describe the distance between graphs with the same spectra but different structure. Sadegh et al. proposed an intelligent method based on the genetic algorithms, with integrating, selecting, and weighting the network features to measure the similarity of complex networks^29^. The complexity of this method depends on the complexity of their feature extraction. However, most methods of network comparison have the problem that the extraction of network information is limited or incomplete thus important structural differences are missed.

Recently, Schieberl proposed a discriminative and computationally efficient differences measure for network comparison^30^. This method has relatively superior polynomial time complexity. More importantly, it can accurately distinguish all the isomorphism and non-isomorphism networks and can quantitatively describe the network differences. It can also compare networks with different sizes. However, this method, regardless of the edge weight, is only applicable to the unweighted networks.

It is generally accepted that weights are coupled in a non-trivial way to the binary network topology, playing an important part in structural organization, functionality and dynamics. For instance, the spreading of emergency diseases in the international airport network is closely related to the number of passengers travelling from one airport to another. In many applications of similarity comparison, such as discriminating between neurological disorders^31^, quantifying changes in temporal evolving network^32^, if these networks are weighted, undoubtedly, more accurate similarity measurement can be obtained after considering the edge weight. Especially, when comparing two weighted complete graphs, like the similarity network between cities obtained by different methods^33^, whose difference mainly comes from the edge weight, and then a dissimilarity metric of weighted networks becomes indispensable.

In view of the above analysis, we propose a quantitative dissimilarity metric for comparing weighted networks based on method proposed by Schieberl^30^. It is assumed that the initial weighted networks are with similarity weights. Firstly, the shortest path lengths are measured through reciprocal edge weights and are rescaled by the ratio of the average shortest path lengths of the weighted network to its binary counterpart. Hence, we can construct a probability matrix based on distance between each pair of nodes, which captures the comprehensive information of the network. Secondly, Jensen-Shannon divergence is used to compare the differences between the distance distribution vectors obtained from probability matrix. Thirdly, the concept and calculation of complementary graph and alpha centrality of weighted network are defined. The quantitative differences between original weighted network and its complementary graph in alpha centrality are respectively computed through Jensen-Shannon divergence. Finally, several synthetic and real-world networks are used to verify the effectiveness and necessity of the proposed WD-metric. Moreover, WD-metric is used to compare original real networks and their skeleton, extracting through Disparity filter and Global Threshold filter when retaining similar edge density, indicating new proposed metric can be used as a criterion for backbone extraction algorithms of complex network.

## Methods

### D-measure

When measuring the difference between two unweighted networks, Schieberl proposed a dissimilarity metric (D-measure), which was defined as a three-term function^30^:

1 $$\begin{aligned} D(G,G')=\omega _{1}\sqrt{\frac{\textit{J}(\mu _{G},\mu _{G'})}{log2}}+ \omega _{2}\left| \sqrt{N\!N\!D(G)}-\sqrt{N\!N\!D(G')}\right| +\frac{\omega _{3}}{2}\left( \sqrt{\frac{\textit{J}(P_{\alpha G},P_{\alpha G'})}{log2}}+\sqrt{\frac{\textit{J}(P_{\alpha G^{c}},P_{\alpha G'^{c}})}{log2}}\right) \end{aligned}$$

where $\omega _{1}$,$\omega _{2}$ and $\omega _{3}$ are arbitrary weights of the terms satisfying $\omega _{1}+\omega _{2}+\omega _{3}=1$. *J* is the *Jensen-Shannon (JS)* divergence.

Instead of comparing vectors whose elements were numbers such as the number of node or edge, average degree and so on, Schieberl considered vectors in which the elements were sets of probability distributions. Particularly, for each node $i=1,2,\ldots ,N$, the node-distance distribution $P_{i}=\{p_i(j)\}$ was defined as the fraction of nodes at distance *j* from node *i*. The set of *N* node-distance distributions $\{P_{1},\ldots ,P_{N}\}$ contains a lot of detailed topological information, such as the degree (number of nodes at distance 1 from *i*) and the closeness centrality (the sum of the inverse distance from *i* to all other nodes). Then, the network node dispersion (NND) was defined as:

2 $$\begin{aligned} N\!N\!D(G)=\frac{\textit{J}(P_{1},\ldots ,P_{N})}{log(d+1)} \end{aligned}$$

where

3 $$\begin{aligned} \textit{J}(P_{1},\ldots ,P_{N})=\frac{1}{N}\sum _{i,j}p_{i}(j)log\left( \frac{p_i(j)}{\mu _{j}}\right) \end{aligned}$$

$\mu _{j}=\sum _{i=1}^{N}p_{i}(j)/N$,*d* is the diameter of network G.

In the first term of Formula (1), averaged connectivity distribution of nodes, $\mu _{G}$ and $\mu _{G'}$ , the set of $\mu _{j}(j=1,2,\ldots ,d)$ and $\mu _{j}'(j=1,2,\ldots ,d')$ were compared, which captured the global topological differences of network G and G’. The second term analyzed the heterogeneity of nodes by comparing the connectivity distribution of each node $P_{i}(i=1,2,\ldots ,N)$ and normalizing by $log(d+1)$. In addition, considering many networks like most *k*-regular networks possess $N\!N\!D=0$, the third term compared the difference values of the graphs and their complements in alpha centrality.

Because of the importance of weight in the research of network structure and function, designing an efficient and quantitative dissimilarity metric applicable to weighted network is very meaningful and necessary. Therefore, we propose the *WD-metric* based on *D-measure*.

### WD-metric

Given the weight, the distances between nodes of weighted network become different real numbers, not just integer any more as in an unweighted network. How to convert them to integers for calculating the node-distance distributions while depicting their meaning of *n-th* order neighbors? In addition, little is known about complement of a weighted network. Moreover, redesigning the reasonable parameter values in calculating alpha centrality of a weighted network is also an important part.

As for the weighted network $G_{\omega }=\langle V_{\omega },E_{\omega }\rangle$, where $V_{\omega }$ and $E_{\omega }$ represent the set of nodes and edges in $G_{\omega }$. Denote *W* as the adjacency matrix of $G_{\omega }$. Here, for consistency of understanding and processing distance, we state that the $\omega _{ij}$ is the similarity weight and the value $\omega _{ij}=0$ if two nodes *i* and *j* are disconnected. In addition, we perform the normalization on weight by dividing the maximum weight. So, the similarity weights are distributed in [0,1].

#### The distance distribution of weighted network

Given a network with similarity weight, the reciprocal of the weight is taken to measure the path length. $L_{\omega }$ is the matrix of shortest path length, whose entry $l_{ij}$ , being the weighted distance from node *i* to node *j*, becomes continuous real number rather than integer. In this case, instead of simply rounding it, we first rescale $L_{\omega }$ through multiplying it by ${\overline{L}} /\overline{L_{\omega }}$ ( $\overline{L_{\omega }}$ and ${\overline{L}}$ are the average shortest path lengths of the weighted network and its binary counterpart, respectively) to get $L_{\omega }'$, and then ceiling the values to get $L_{\omega }''$. By doing this, the original real distances are classified thus we can count up the numbers of nodes with the same distance from node *i* and then divide them by $N-1$ to obtain the node-distance distributions of weighted network $P_{i}^{\omega }=\{p_{i}^{\omega }(j)\}(i=1,2,\ldots ,N)$. Most importantly, the method of rescaling distance can retain the topological properties about *n-th* order neighbor. The set of *N* node-distance distribution $\{P_{1}^{\omega },P_{2}^{\omega },\ldots ,P_{N}^{\omega } \}$ forms a matrix $P_{\omega }$ with the element $p_{i}^{\omega }(j)$ being the fraction of nodes that are connected to the node *i* at distance *j*, similar to the case for unweighted network. In particular, the matrix $P_{\omega }$ includes one column for those disconnected nodes. Therefore, our method can also work well for the disconnected networks. See Supplementary Note 1 for detailed description with a simple example.

#### Complement of weighted network

There is very little discussion on the complement of a weighted network. We give a similar and reasonable definition of the complementary graph of a weighted network referring to the complement of an unweighted network.

For an unweighted network *G* with adjacency matrix *A(G)* , its complementary graph $G^{c}$ , in the matrix representation, can be denoted as $A(G^{c})=K_{n}-A(G)$. $K_{n}$ is a matrix whose entries are all equal to one.

For a weighted network $G_{\omega }$, with similarity weights distributed in [0,1], denoting its adjacency matrix as $W(G_{\omega })$, correspondingly, its complementary graph can be defined as $W(G_{\omega }^{c})= K_{n}-W(G_{\omega })$, where $K_{n}$ is a matrix whose entries are all equal to one.

#### Alpha centrality

Since alpha centrality considers not only the interaction between nodes, but also the information of each node that are independent of others^34^, it is widely studied as an important property of network. It is generally formed as:

4 $$\begin{aligned} x=\alpha Ax+\beta \end{aligned}$$

where *A* is the adjacency matrix of network *G*, $\alpha$ is the attenuation factor and $\beta$ is an exogenous factor vector. It can be proved that the solution of equation converges for $\alpha <1/\lambda _{max}$ , where $\lambda _{max}$ is the spectral radius of the network.

According to the Perron–Frobenius theory, in a real symmetric matrix *M* , $\lambda _{max}\le {max}_{i}\sum _{j}M_{ij}$. Therefore, in a graph, $\lambda _{max}$ must be less than the maximum degree. Schieberl set $\alpha =1/N$ and considered link density of every node as an exogenous factor vector for an unweighted network. In a weighted network $G_{\omega }$ , the adjacency matrix *W* is also symmetric, then $\lambda _{max}$ is bounded from above by the maximum node strength. Because the weights of $G_{\omega }$ are distributed in [0,1], the maximum node strength is bounded from above by *N*. Hence, we set $\alpha =1/N$, $\beta =S/[(N-1)\cdot {\overline{\omega }}]$, where ${\overline{\omega }}$ is the average weight, *S* is the node strength vector.

As known, *JS* divergence is often used to measure the difference between two probability distributions. Therefore, when considering the influence of alpha centrality, we process the calculated alpha centrality vector $V_{\alpha }$ to obtain $P_{\alpha }$ who is a discrete probability distribution with one dimension more than $V_{\alpha }$:

5 $$\begin{aligned} P_{\alpha }=\frac{1}{N}\left[ V_{\alpha },N-\sum _{i=1}^{N}\left( V_{\alpha }(i)\right) \right] \end{aligned}$$

#### Expression of the WD-metric

Considering the effects of global and local features, we can obtain a few related vectors based on the above definitions of the distance probability matrix, complementary graph and alpha centricity of a weighted network.

First of all, through the distance probability matrix $P_{\omega }$, we can obtain the average proportion of each order neighbors:

6 $$\begin{aligned} \mu _{j}^{\omega }=\sum _{i=1}^{N}p_{i}^{\omega }(j)/N \end{aligned}$$

Further, we can calculate the value of node dispersion of weighted network (WNND), which is defined as:

7 $$\begin{aligned} W\!N\!N\!D(G)=\frac{\textit{J}(P_{1}^{\omega },P_{2}^{\omega },\ldots ,P_{N}^{\omega })}{log(m+1)} \end{aligned}$$

with

8 $$\begin{aligned} \textit{J}(P_{1}^{\omega },P_{2}^{\omega },\ldots ,P_{N}^{\omega })=\frac{1}{N}\sum _{i,j}p_{i}^{\omega }(j)log\left( \frac{p_{i}^{\omega }(j)}{\mu _{j}^{\omega }}\right) \end{aligned}$$

where *m* is the number of columns of the distance probability matrix $P_{\omega }$ , and *J* is the *JS* divergence.

Finally, the quantitative dissimilarity metric of weighted network is proposed as:

9 $$\begin{aligned} W\!D(G_{1}^{\omega },G_{2}^{\omega })= & {} \omega _{1}\sqrt{\frac{\textit{J}(\mu _{G_{1}^{\omega }},\mu _{G_{2}^{\omega }})}{log2}} +\omega _{2}\left| \sqrt{W\!N\!N\!D(G_{1}^{\omega })}-\sqrt{W\!N\!N\!D(G_{2}^{\omega })}\right| \nonumber \\&+\frac{\omega _{3}}{2}\left( \sqrt{\frac{\textit{J}(P_{\alpha G_{1}^{\omega }},P_{\alpha G_{2}^{\omega }})}{log2}}+\sqrt{\frac{\textit{J}(P_{\alpha {G_{1}^{\omega }}^{c}},P_{\alpha {G_{2}^{\omega }}^{c}})}{log2}}\right) \end{aligned}$$

Here we set the weights $\omega _{1}=\omega _{2}=0.45$ and $\omega _{3}=0.1$ as Schieberl did to quantify structural dissimilarities between weighted networks. On one hand, considering of the consistency, we hope that the weighted dissimilarity metric is still applicable to the unweighted network. On the other hand, the weights here respectively represent the influence of networks global (first term), networks local (second term) features and the network heterogeneity (third term) on the network differences. The value of each term of the WD-metric supposed to be proportional to that of unweighted. We calculate several pairs of real networks and get basically consistent results.

**Figure 1:**
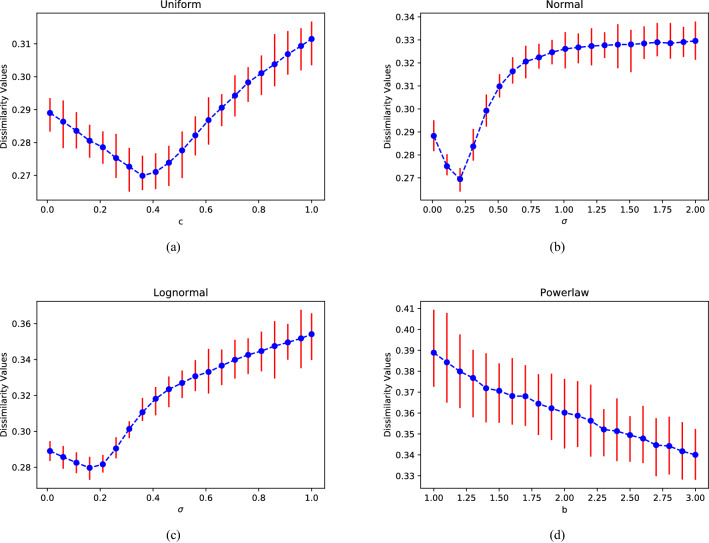
Comparisons between complete graphs with different weights or not. The weights are drawn from (**a**) Uniform distribution $U[1-c,1+c](0\le c\le 1)$; (**b**) Normal distribution $X\sim N(1,\sigma ^{2})$; (**c**) Lognormal distribution $lnX\sim N(\mu ,\sigma ^{2})$; (**d**) Power-law distribution $f(x,b)=b/x^{b+1}$ .

## Results

Leveraging the WD-metric we propose, several groups of experiments are performed on synthetic networks and real networks to verify the necessity and validity of new proposed metric. Note that, if no specific instructions in this paper, the dissimilarity values (D-values) between all synthetic networks are average results of running 100 times, and the size of synthetic network is N=100.

### Complete graphs with four edge weight distributions

**Figure 2:**
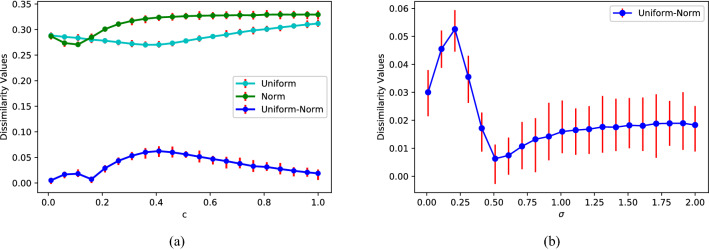
Comparisons between weighted complete graphs. (**a**) The cyan and the green lines depict the differences between weighted complete graphs and its binary counterpart, weights drawn from the uniform distribution $U[1-c,1+c]$ and the normal distribution $N(1,\sigma ^{2}),(\sigma =2c)$, respectively. The blue line depicts the difference of two weighted networks with the weights drawn from $U[1-c,1+c]$ and $N(1,\sigma ^{2}),(\sigma =2c)$. (**b**) The differences of two weighted networks with the weights drawn from *U*[0, 2] and $N(1,\sigma ^{2})$ change with $\sigma$.

In order to verify the effectiveness of the WD-metric in comparison between diverse weighted networks, the weights drawn from different distributions are first added to the complete graphs, and then the dissimilarity values (D-values) between the complete graphs with and without weights are calculated and shown in Figs. 1 and 2.

As shown in Fig. 1, there is a significant difference between before and after weighting on a complete graph. Meanwhile the D-values change gradually with the corresponding parameters under different weighting modes. They indicate that our method captures the influence of the weight on the network structure. Except the comparison between a weighted and an unweighted network, we also compare the difference between two weighted networks. As red lines shown in Fig. 2, the D-values between two networks with same topology but different weights are relatively small, but they still change significantly with the weight, which further indicates the WD-metric effectively depicts the effect of weight on the network.

### Incomplete graphs with different edge densities

Having observed the differences between weighted complete graphs, we would like to see the performance of the WD-metric on the weighted incomplete graphs. Therefore, we use the WD-metric to observe the differences before and after weighting on *Erdos–Renyi* (*ER*) network and *Barabasi–Albert* (*BA*) network with different densities.

**Figure 3:**
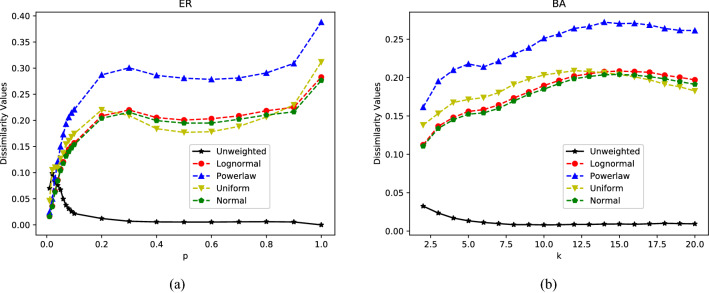
Comparison between incomplete graphs with different edge densities. The black curves depict the differences between two unweighted networks with the same density change with the probability *p* of connecting pairs of nodes or average degree *k*. Other colored curves show the differences between weighted network and its binary counterpart at various densities. The edge weights are drawn from lognormal distribution $(E(x)=1,\sigma =0.1)$, power-law distribution $(b=1)$, uniform distribution *U*[0, 2] and normal distribution $(\mu =1,\sigma =0.1)$. (**a**) The difference between *Erdos–Renyi* (*ER*) networks; (**b**) The difference between *Barabasi–Albert* (*BA*) networks.

As shown of the black curves in Fig. 3, there is little difference between two unweighted networks (UD-values) at any of the same density. However, the colored curves show that the difference after weighting (WD-values) increase obviously in most cases, except on ER network with small *p*. The possible reason may be that small connecting probability causes the ER network to be divided into many disconnected groups, so the UD-values are relatively larger. Moreover, in this case, a small quantity of edge weight has little effect on network, so there is no clear difference between UD-values and WD-values. In addition, from the colored curves, it is not difficult to find that the WD-values wholly increase with the increasing of the edge density. That is, when the network is sparse, the weight has little impact on the structure, while in the dense network, the weight has a greater impact. These results are quite consistent with what we know, which further represents the effectiveness and feasibility of our proposed WD-metric.

### Comparison between neural networks

**Figure 4:**
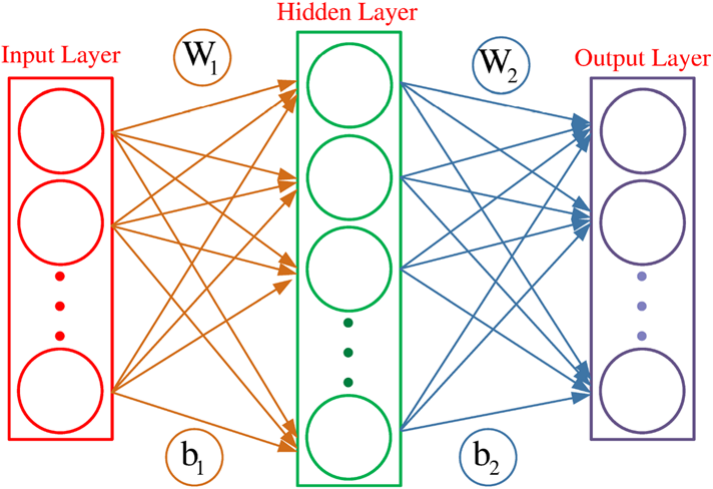
A simplified two-layer neural network. The circles represent the neurons at each layer of the neural network, corresponding to the nodes in the complex network. The correlations between neurons correspond to edges, and the different feedback intensities between neurons correspond to edge weights in the complex network.

As an interdisciplinary technology, neural network has been widely used in various fields to tackle the problems like classification and prediction in recent years^35^. Figure 4 shows a simplified two-layer neural network, composed of many neurons from input layer, hidden layer and output layer, and weighted edges. Neural network is a typical weighted network with specific functions. By continually training data and adjusting edge weights, the new neural network usually has better ability in prediction or classification. We try to use WD-metric to compare these neural networks with different prediction or classification accuracy. If the accuracy of two neural networks is closer, and the dissimilarity between them is smaller, it will further probe the validity of the WD-metric in capturing the function of weighted networks.

Here, we perform some experiments on the classical BP neural network for pattern recognition of handwritten numbers. By inputting 4 groups of training sets with size of 10, 100, 1000 and 10,000, we can obtain four neural networks with different weights but the same topology connection mode. Then, WD-metric is used to compare these networks.

**Table 1:** The D-values for each pair of neural networks obtained by different sizes of training sets. The number of hidden layers is 30 and training times is 100. Number in parentheses represents the classification accuracy of the corresponding neural network. The larger the training set, the higher the accuracy.

The size of training sets (accuracy)	10 (19%)	100 (53%)	1000 (79%)	10,000 (97%)
10	0	0.0042	0.0119	0.0521
100	0.0042	0	0.0107	0.0509
1000	0.0119	0.0107	0	0.0402
10,000	0.0521	0.0509	0.0402	0

Table 1 shows that when the sizes of training sets are different, WD-metric can capture the differences between corresponding neural networks with different classification ability. D-values increase gradually between network with 10 training sets and networks with training sets 100, 1000 and 10,000, while D-values decrease gradually between network with 10,000 training sets and networks with training sets 10,100 and 1000. This shows when the difference of classification accuracy of networks is larger, the D-value between them is larger. The results further manifest that the WD-metric is quantitative and effective for measuring the distance between networks with different functions caused by weights.

### Distances between real weighted networks

**Figure 5:**
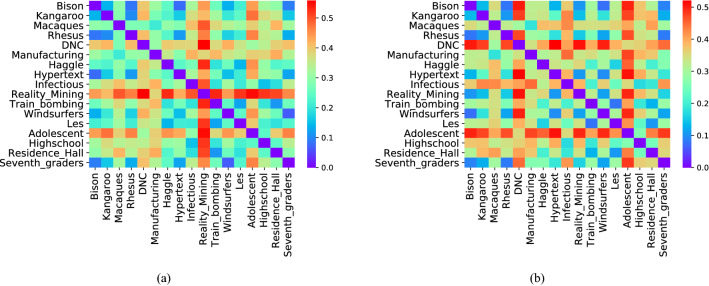
Difference between more weighted real networks. (**a**) Heatmap of the dissimilarity values for each pair of weighted real networks; (**b**) The difference between the real networks when ignoring their weights.

After the comparison between synthetic networks, in order to observe the performance of the WD-metric on real-world networks, we make pair-by-pair comparison among various weighted real networks and the results are shown in Fig. 5a.

**Table 2:** The basic statistics of the real networks. These 17 weighted networks include 4 types: animal, online communication, human contact and human social. |V|, |E|, $\langle$k$\rangle$, $\langle$s$\rangle$ represents the number of nodes, the number of edges, average degree, and average strength of network, respectively.

Network	Directed	|N|	|E|	$\langle$k$\rangle$	$\langle$s$\rangle$
**Animal**					
Bison	Yes	26	314	24.15	69
Kangaroo	No	17	91	10.71	65.29
Macaques	Yes	62	1187	38.29	78.55
Rhesus	Yes	16	111	13.88	80.88
**Online communication**					
DNC	Yes	2029	5598	5.44	36.89
Manufacturing	Yes	167	5784	69.26	992
**Human contact**					
Haggle	No	274	2899	15.50	124.21
Hypertext	No	113	2196	38.87	368.46
Infectious	No	410	2765	13.49	84.38
Reality mining	No	96	2539	52.90	22,633.42
Train bombing	No	64	243	7.59	8.81
Windsurfers	No	43	336	15.63	56.09
Les	No	77	254	6.60	21.30
**Human social**					
Adolescent	Yes	2539	12,969	10.22	29.71
Highschool	Yes	70	366	10.46	14.46
Residence hall	Yes	217	2672	24.63	83.21
Seventh graders	Yes	29	376	25.93	51.03

17 data sets of 4 networks types: Animal, Online Communication, Human Contact and Human Social, are considered. Table 2 shows the basic statistics of them. All networks here presented are freely available at The Koblenz Network Collection (http://konect.uni-koblenz.de/). We also calculate the differences between those networks when ignoring the weight, and the results shown in Fig. 5b. It can be found that there is a significant difference between the two figures. What’s more, as shown in Fig. 5a, the dissimilarities between Reality Mining and other networks are very large under consideration of weight. If not, shown as Fig. 5b, Reality Mining is submerged in the networks, which further indicates the necessity of designing the dissimilarity metric of weighted network. Moreover, we can find that the similarity between some networks with the same type are higher, such as Animal. However, some networks with the same type, such as Human Contact, are also quite different from each other, especially the dissimilarities between Reality Mining and other same type networks are very large. Probably because the classification of networks only by their domain is not enough. See Supplementary Note 3 for specific dissimilarity values between various real networks.

### Application of the WD-metric to backbones extraction

In a large-scale network, the extraction of truly relevant nodes or connections forming the network’s backbone can help form reduced but meaningful representations of a large-scale complex network, and understand its fundamental structure and function^36^. However, many existing extraction methods are mainly for retaining one or more topological attributes. For example, the classical method of Disparity filter proposed by Serrano^37^, still qualitatively shown its superiority to the global threshold filter mainly through the heterogeneity of the weight distribution.

**Figure 6:**
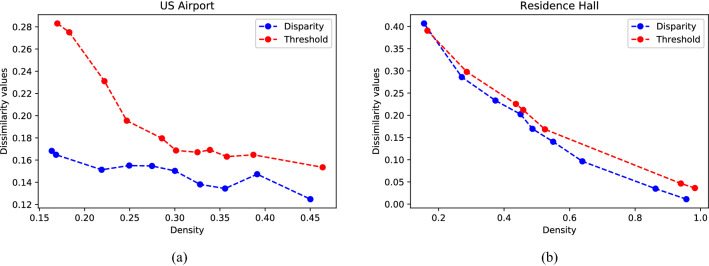
D-values between the filtered backbones and the original networks. (**a**) For the U.S. Airport. (**b**) For the Residence Hall. The abscissa represents the edge density proportion of extraction backbone to the original network.

However, our proposed WD-metric can quantitatively measure the dissimilarity of weighted network from comprehensive information. Figure 6 presents us the D-values between the U.S. Airport and Residence Hall network and their backbones. On one hand, with the increase of edge density, D-values gradually decrease as a whole, which can’t agree more about the fact that the subgraph with lager density retains more information. On the other hand, the blue line is almost below the red line, quantitatively and intuitively indicating the disparity filter is superior to the global threshold filter. The WD-metric can be used as a criterion for backbone extraction algorithms of complex network.

## Discussion

In this paper, we propose a qualitative dissimilarity metric applicative to weighted networks (WD-metric) based on the method of D-measure^30^ only for unweighted networks. Especially, for disconnected networks, it also performs well. Various experiments have shown that WD-metric can capture the influence of the weight on the network structure, and quantitatively and effectively measure the dissimilarity of weighted networks. In addition, it can depict the influence of edge density on network structure. On one hand, when the network is sparse, the weight has little impact on the structure. On the other hand, while in the dense network, the weight has a greater impact. Furthermore, the WD-metric can be used as a criterion for backbone extraction algorithms of complex network.

We have compared among some real-world networks and obtained the dissimilarity values between them through the WD-metric but without further analyzing the practical significance of the dissimilarity values. Scholars from different fields can use it combined with various practical problems yield interesting results and applications. Moreover, from the perspective of minimizing D-value between original network and its backbone, developing a new method of backbone extraction is a meaningful idea. In addition, we can pay more attention to the relationship between network differences and network functionalities such as the percolation and spreading dynamics. How to set the weight of each term of the WD-metric is also worth seriously considering.

## Supplementary information

Supplementary material 1 (pdf 1189 KB)

## Declarations

### Code availability

All data generated and analysed in the manuscript are reproducible based on the algorithms detailed in the article (see the “Methods” and the “Results” sections).
