# Supplementary material for: Characterizing dissimilarity of weighted networks
Source: Sci Rep. 2021 Mar 11;11:5768. doi: 10.1038/s41598-021-85175-9 (PMC7952696; doi:10.1038/s41598-021-85175-9)
Supplement: Supplementary file 1 — Supplementary material 1 (pdf 1189 KB) [file 41598_2021_85175_MOESM1_ESM.pdf]

Characterizing dissimilarity of weighted networks

Yuanxiang Jiang[1], Meng Li[1], Ying Fan[1], and Zengru Di[1,*]

[1]Beijing Normal University, School of Systems Science, Beijing, 100875, China

***zdi@bnu.edu.cn**

SUPPLEMENTARY NOTE 1: A DETAILED EXAMPLE OF CONSTRUCTION OF THE DISTANCE PROBABILITY MATRIX

For a better understanding of how the distance probability matrix of a weighted network is constructed, we give a concrete example.

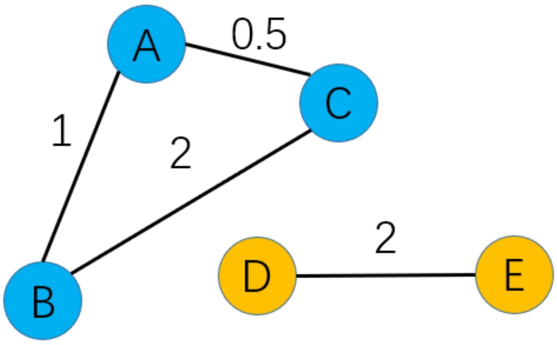

Supplementary Figure 1: A particular network as an example

Given a weighted network G shown in Supplementary Figure 1, whose weights are similarity weight and are marked on the edges. Denote the network as an adjacency matrix W_0.

(1) Normalization: $W = \dfrac{W_0}{\max(W_0)}$

$$W_0 = \begin{pmatrix} 0 & 1 & 1/2 & 0 & 0 \\ 1 & 0 & 2 & 0 & 0 \\ 1/2 & 2 & 0 & 0 & 0 \\ 0 & 0 & 0 & 0 & 2 \\ 0 & 0 & 0 & 2 & 0 \end{pmatrix} \xrightarrow{Normalization} W = \begin{pmatrix} 0 & 1/2 & 1/4 & 0 & 0 \\ 1/2 & 0 & 1 & 0 & 0 \\ 1/4 & 1 & 0 & 0 & 0 \\ 0 & 0 & 0 & 0 & 1 \\ 0 & 0 & 0 & 1 & 0 \end{pmatrix}$$

(2)Taking the reciprocals of edge weights to transform them into dissimilarity weights: $W_{ii}' = 0$ and

$$W'_{ij(i \neq j)} = \begin{cases} \dfrac{1}{W_{ij}}, (W_{ij} \neq 0) \\ \infty, (W_{ij} = 0) \end{cases}$$

$$W=\begin{pmatrix} 0 & 1/2 & 1/4 & \infty & \infty \\ 1/2 & 0 & 1 & \infty & \infty \\ 1/4 & 1 & 0 & \infty & \infty \\ \infty & \infty & \infty & 0 & 1 \\ \infty & \infty & \infty & 1 & 0 \end{pmatrix} \xrightarrow{Reciprocal} W'=\begin{pmatrix} 0 & 2 & 4 & \infty & \infty \\ 2 & 0 & 1 & \infty & \infty \\ 4 & 1 & 0 & \infty & \infty \\ \infty & \infty & \infty & 0 & 1 \\ \infty & \infty & \infty & 1 & 0 \end{pmatrix}$$

(3) Calculating the weighted path length with dissimilarity weight and getting the shortest path length for any pair of nodes by *Dijkstra* algorithm. The corresponding matrix of shortest path length L_ω for weighted network and L for its unweighted counterpart are as follows:

$$L_\omega=\begin{pmatrix} 0 & 2 & 3 & \infty & \infty \\ 2 & 0 & 1 & \infty & \infty \\ 3 & 1 & 0 & \infty & \infty \\ \infty & \infty & \infty & 0 & 1 \\ \infty & \infty & \infty & 1 & 0 \end{pmatrix} \text{ and } L=\begin{pmatrix} 0 & 1 & 1 & \infty & \infty \\ 1 & 0 & 1 & \infty & \infty \\ 1 & 1 & 0 & \infty & \infty \\ \infty & \infty & \infty & 0 & 1 \\ \infty & \infty & \infty & 1 & 0 \end{pmatrix}$$

(4) Calculating the average shortest path lengths of the weighted network $\overline{L_\omega}$ and average shortest path lengths of its unweighted counterpart $\overline{L}$. In a disconnected network, only accessible paths are considered. For Supplementary Fig.1: $\overline{L_\omega}=\frac{7}{4},\overline{L}=1$

(5) Rescaling to get $L_\omega'=L_\omega\times\frac{\overline{L}}{\overline{L_\omega}}$ and then Ceiling: $L_\omega''=[L_\omega']+1$ to get L_ω''.

$$L_\omega=\begin{pmatrix} 0 & 2 & 3 & \infty & \infty \\ 2 & 0 & 1 & \infty & \infty \\ 3 & 1 & 0 & \infty & \infty \\ \infty & \infty & \infty & 0 & 1 \\ \infty & \infty & \infty & 1 & 0 \end{pmatrix} \xrightarrow{Rescaling} L_\omega'=\begin{pmatrix} 0 & 8/7 & 12/7 & \infty & \infty \\ 8/7 & 0 & 4/7 & \infty & \infty \\ 12/7 & 4/7 & 0 & \infty & \infty \\ \infty & \infty & \infty & 0 & 4/7 \\ \infty & \infty & \infty & 4/7 & 0 \end{pmatrix} \xrightarrow{Ceiling} L_\omega''=\begin{pmatrix} 0 & 2 & 2 & \infty & \infty \\ 2 & 0 & 1 & \infty & \infty \\ 2 & 1 & 0 & \infty & \infty \\ \infty & \infty & \infty & 0 & 1 \\ \infty & \infty & \infty & 1 & 0 \end{pmatrix}$$

(6) Counting up the numbers of nodes with the same distance from node *i,* to obtain the matrix *Num.* Counting: $Num(i,j')=\sum_{j=1}^{N} I(L_\omega''(i,j)),(i,j=1,2,\cdots,N),(j'=1,2,\cdots,d+1),d=\max(L_\omega''(i,j)\neq\infty))$. If $L_\omega''(i,j)=j'$, $I(x)=1$; otherwise, $I(x)=0$, and then to obtain the matrix of distance probability distributions $P_\omega=\frac{Num}{N-1}$ of weighted network . The last column denotes the proportion of disconnected nodes.

$$L_\omega''=\begin{pmatrix} 0 & 2 & 2 & \infty & \infty \\ 2 & 0 & 1 & \infty & \infty \\ 2 & 1 & 0 & \infty & \infty \\ \infty & \infty & \infty & 0 & 1 \\ \infty & \infty & \infty & 1 & 0 \end{pmatrix} \xrightarrow{Counting} Num=\begin{pmatrix} 0 & 2 & 2 \\ 1 & 1 & 2 \\ 1 & 1 & 2 \\ 1 & 0 & 3 \\ 1 & 0 & 3 \end{pmatrix} \xrightarrow{Dividing} P_\omega=\begin{pmatrix} 0 & 0.5 & 0.5 \\ 0.25 & 0.25 & 0.5 \\ 0.25 & 0.25 & 0.5 \\ 0.25 & 0 & 0.75 \\ 0.25 & 0 & 0.75 \end{pmatrix}$$

SUPPLEMENTARY NOTE 2: THE WEIGHT NORMALIZATION ANALYSIS

In the calculation of dissimilarity between weighted networks, we normalize the edge weights by dividing the maximum. Because that, in the third term of WD-metric, we define the complementary graph of weighted network. After weight normalization, the definition is a good generalization from unweighted to weight network. However, the maximum weight is stochastic for the different distributions. Whether performing the weight normalization or the way of normalization caused the results? Therefore, we also execute the experiment without normalization and put relevant results in the Supplementary Figure 3. By comparing Supplementary Figures 2 and 3, it is not difficult to find that normalization on edge weight by dividing the maximum does not change the qualitative conclusion: Different weighting modes cause different dissimilarity curves.

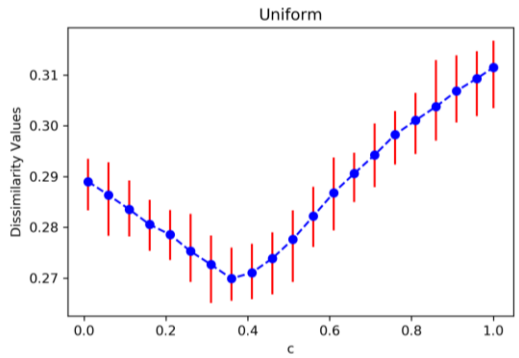

a

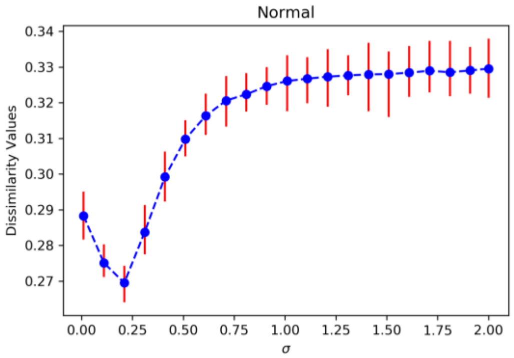

b

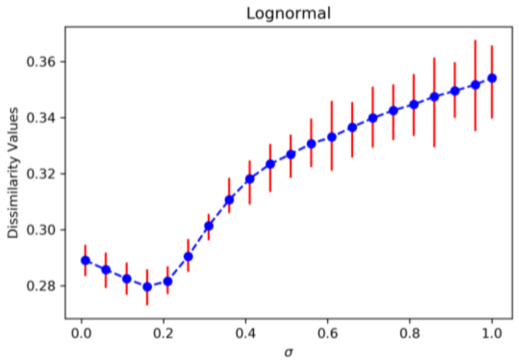

c

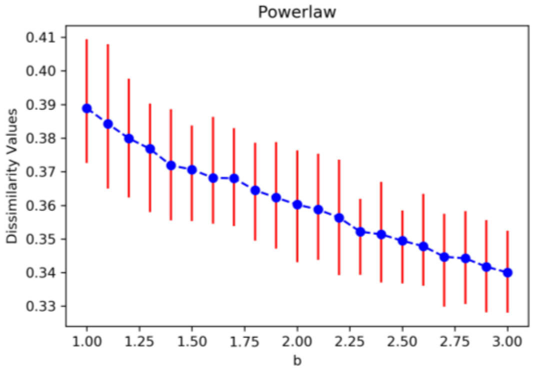

d

Supplementary Figure 2: Comparisons between complete graphs with normalized weights. The weights are drawn from (a) Uniform distribution $U[1-c,1+c](0 \le c \le 1)$; (b) Normal distribution $X \sim N(1,\sigma^2)$; (c) Lognormal distribution $lnX \sim N(\mu,\sigma^2)$;(d) Power-law distribution $f(x,b) = b/x^{b+1}$.

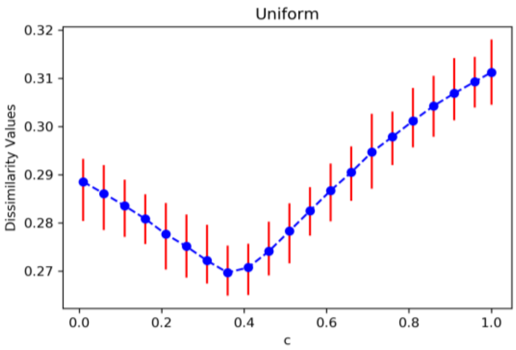

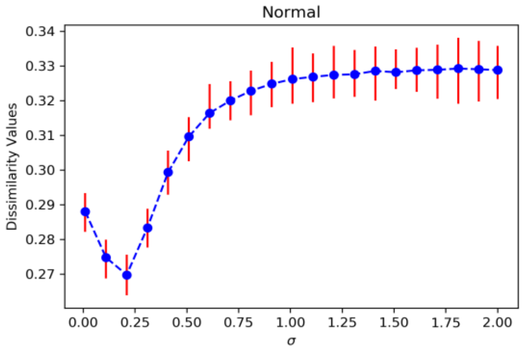

a **b**

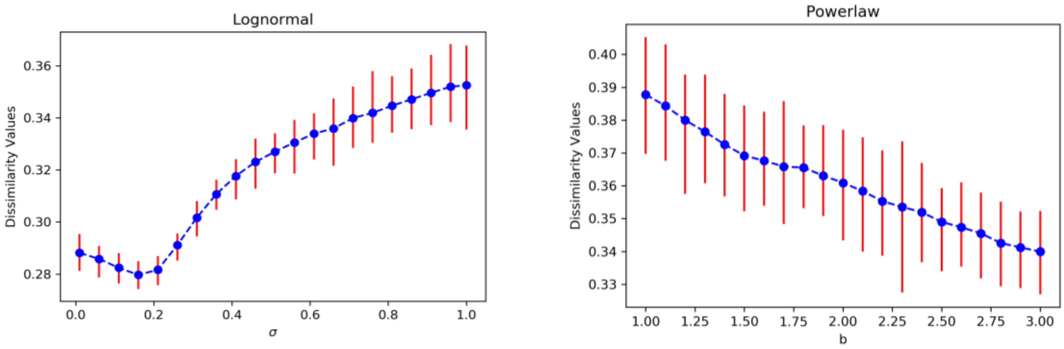

c **d**

Supplementary Figure 3: Comparisons between complete graphs with weights without being normalized. The weights are drawn from (a) Uniform distribution $U[1-c, 1+c](0 \leq c \leq 1)$; (b) Normal distribution $X \sim N(1, \sigma^2)$; (c) Lognormal distribution $lnX \sim N(\mu, \sigma^2)$;(d) Power-law distribution $f(x, b) = b/x^{b+1}$.

SUPPLEMENTARY NOTE 3: DISSIMILARITY VALUES BETWEEN REAL DATASETS

Supplementary Table 1. Specific dissimilarity values between 17 data sets of 4 networks types

Network Name	Adolescent	Highschool	Residence_Hall	Bison	Rhesus	Seventh_graders	Macaques	Hypertext	Windsurfers	Manufacturing	Kangaroo	Reality_Mining	Infectious	DNC	Train_bombing	Les	Haggle
Adolescent	0	0.2390	0.3963	0.4386	0.4626	0.4627	0.3894	0.4277	0.4445	0.3899	0.4767	0.5490	0.2985	0.3128	0.3785	0.3946	0.4484
Highschool	0.2390	0	0.2406	0.3187	0.3516	0.3433	0.3373	0.3130	0.3158	0.3843	0.3738	0.5195	0.1545	0.3404	0.1985	0.2202	0.3042
Residence_Hall	0.3963	0.2406	0	0.2989	0.3344	0.2908	0.4168	0.3003	0.2580	0.4267	0.3516	0.5090	0.1909	0.3885	0.1121	0.1919	0.2328
Bison	0.4386	0.3187	0.2989	0	0.0859	0.0932	0.2974	0.0770	0.1355	0.3128	0.1408	0.4401	0.3245	0.3979	0.2594	0.2150	0.2421
Rhesus	0.4626	0.3516	0.3344	0.0859	0	0.1336	0.2767	0.1175	0.1719	0.3020	0.1037	0.4657	0.351	0.4073	0.2983	0.2121	0.234
Seventh_graders	0.4627	0.3433	0.2908	0.0932	0.1336	0	0.3180	0.1247	0.0664	0.3342	0.1610	0.4556	0.3454	0.4062	0.2622	0.2300	0.2444
Macaques	0.3894	0.3373	0.4168	0.2974	0.2767	0.3180	0	0.2821	0.3374	0.2074	0.2853	0.4952	0.4053	0.2763	0.3772	0.3145	0.3010
Hypertext	0.4277	0.3130	0.3003	0.077	0.1175	0.1247	0.2821	0	0.1509	0.2880	0.1635	0.4247	0.3224	0.3846	0.2667	0.2109	0.2395
Windsurfers	0.4445	0.3158	0.2580	0.1355	0.1719	0.0664	0.3374	0.1509	0	0.3608	0.2032	0.4418	0.3193	0.4224	0.2440	0.2433	0.2612
Manufacturing	0.3899	0.3843	0.4267	0.3128	0.3020	0.3342	0.2074	0.2880	0.3608	0	0.2730	0.3812	0.4201	0.3057	0.4132	0.3433	0.3507
Kangaroo	0.4767	0.3738	0.3516	0.1408	0.1037	0.1610	0.2853	0.1635	0.2032	0.2730	0	0.4106	0.3703	0.4139	0.3184	0.2410	0.2275
Reality_Mining	0.549	0.5195	0.5090	0.4401	0.4657	0.4556	0.4952	0.4247	0.4418	0.3812	0.4106	0	0.4971	0.5575	0.5301	0.5185	0.5282
Infectious	0.2985	0.1545	0.1909	0.3245	0.3510	0.3454	0.4053	0.3224	0.3193	0.4201	0.3703	0.4971	0	0.3795	0.1940	0.2061	0.2720
DNC	0.3128	0.3404	0.3885	0.3979	0.4073	0.4062	0.2763	0.3846	0.4224	0.3057	0.4139	0.5575	0.3795	0	0.3752	0.3584	0.3945
Train_bombing	0.3785	0.1985	0.1121	0.2594	0.2983	0.2622	0.3772	0.2667	0.2440	0.4132	0.3184	0.5301	0.1940	0.3752	0	0.1262	0.1944
Les	0.3946	0.2202	0.1919	0.2150	0.2121	0.2300	0.3145	0.2109	0.2433	0.3433	0.2410	0.5185	0.2061	0.3584	0.1262	0	0.1436
Haggle	0.4484	0.3042	0.2328	0.2421	0.2340	0.2444	0.3010	0.2395	0.2612	0.3507	0.2275	0.5282	0.2720	0.3945	0.1944	0.1436	0

Supplementary Table 2. Specific dissimilarity values between 17 real networks when ignoring their weights

Network Name	Adolescent	Highschool	Residence_Hall	Bison	Rhesus	Seventh_graders	Macaques	Hypertext	Windsurfers	Manufacturing	Kangaroo	Reality_Mining	Infectious	DNC	Train_bombing	Les	Haggle
**Adolescent**	0	0.2654	0.4328	0.4971	0.4688	0.4832	0.4008	0.5077	0.4930	0.4738	0.4740	0.4942	0.3512	0.3495	0.4060	0.4189	0.4532
**Highschool**	0.2654	0	0.2492	0.3736	0.3403	0.3642	0.3217	0.3974	0.3646	0.3875	0.3765	0.3853	0.1714	0.3610	0.1901	0.2257	0.3179
**Residence_Hall**	0.4328	0.2492	0	0.3104	0.3251	0.3553	0.4082	0.3301	0.3032	0.3711	0.3846	0.3402	0.188	0.4047	0.1303	0.1065	0.2029
**Bison**	0.4971	0.3736	0.3104	0	0.0796	0.0896	0.3482	0.1313	0.0755	0.3225	0.1369	0.1050	0.3673	0.5031	0.2966	0.3179	0.3233
**Rhesus**	0.4688	0.3403	0.3251	0.0796	0	0.0837	0.3248	0.1788	0.1164	0.2943	0.1072	0.1514	0.3850	0.4715	0.2581	0.2775	0.2852
**Seventh_graders**	0.4832	0.3642	0.3553	0.0896	0.0837	0	0.2955	0.1886	0.1168	0.2654	0.1242	0.1616	0.4150	0.4471	0.2868	0.2891	0.2667
**Macaques**	0.4008	0.3217	0.4082	0.3482	0.3248	0.2955	0	0.3753	0.3456	0.1815	0.3218	0.3471	0.4257	0.2947	0.3421	0.3602	0.3815
**Hypertext**	0.5077	0.3974	0.3301	0.1313	0.1788	0.1886	0.3753	0	0.0982	0.3230	0.2238	0.1074	0.3888	0.5199	0.3245	0.3393	0.3371
**Windsurfers**	0.4930	0.3646	0.3032	0.0755	0.1164	0.1168	0.3456	0.0982	0	0.3061	0.1825	0.1143	0.3646	0.4983	0.2829	0.3033	0.3016
**Manufacturing**	0.4738	0.3875	0.3711	0.3225	0.2943	0.2654	0.1815	0.3230	0.3061	0	0.3249	0.3314	0.4484	0.3392	0.3469	0.3387	0.3090
**Kangaroo**	0.4740	0.3765	0.3846	0.1369	0.1072	0.1242	0.3218	0.2238	0.1825	0.3249	0	0.1414	0.4278	0.4786	0.3184	0.3236	0.3342
**Reality_Mining**	0.4942	0.3853	0.3402	0.105	0.1514	0.1616	0.3471	0.1074	0.1143	0.3314	0.1414	0	0.3894	0.5094	0.3225	0.3404	0.3536
**Infectious**	0.3512	0.1714	0.1880	0.3673	0.3850	0.4150	0.4257	0.3888	0.3646	0.4484	0.4278	0.3894	0	0.4050	0.2034	0.2394	0.3176
**DNC**	0.3495	0.3610	0.4047	0.5031	0.4715	0.4471	0.2947	0.5199	0.4983	0.3392	0.4786	0.5094	0.405	0	0.3966	0.3913	0.4009
**Train_bombing**	0.406	0.1901	0.1303	0.2966	0.2581	0.2868	0.3421	0.3245	0.2829	0.3469	0.3184	0.3225	0.2034	0.3966	0	0.0782	0.2065
**Les**	0.4189	0.2257	0.1065	0.3179	0.2775	0.2891	0.3602	0.3393	0.3033	0.3387	0.3236	0.3404	0.2394	0.3913	0.0782	0	0.1606
**Haggle**	0.4532	0.3179	0.2029	0.3233	0.2852	0.2667	0.3815	0.3371	0.3016	0.3090	0.3342	0.3536	0.3176	0.4009	0.2065	0.1606	0
